# Enhanced Motivational Modulation of Motor Behaviour with Subthalamic Nucleus Deep Brain Stimulation in Parkinson's Disease

**DOI:** 10.1155/2019/3604372

**Published:** 2019-01-02

**Authors:** Maja Kojovic, Andrea Higgins, Pablo Mir, Marjan Jahanshahi

**Affiliations:** ^1^Department of Neurology, University of Ljubljana, Ljubljana, Slovenia; ^2^Sobell Department of Motor Neuroscience & Movement Disorders, UCL Institute of Neurology, London, UK; ^3^Department of Clinical Psychology, Trinity College Dublin, Dublin, Ireland; ^4^Department of Clinical Psychology, University College Dublin, Dublin, Ireland; ^5^Unidad de Trastornos del Movimiento, Servicio de Neurología y Neurofisiología Clínica, Instituto de Biomedicina de Sevilla, Hospital Universitario Virgen del Rocío/CSIC/Universidad de Sevilla, Seville, Spain; ^6^Centro de Investigación Biomédica en Red Sobre Enfermedades Neurodegenerativas (CIBERNED), Cáceres, Spain; ^7^The Clinical Hospital of Chengdu Brain Science Institute, MOE Key Lab for Neuroinformation, University of Electronic Science and Technology of China, Chengdu, China

## Abstract

**Background:**

Motivational improvement of movement speed in Parkinson's disease (PD) is observed in life-threatening situations and has been empirically demonstrated in experimental studies using reaction time paradigms.

**Objectives:**

To address two clinically relevant questions: first, if in PD, motivational modulation through provision of monetary incentive on a sorting task that approximates performance on everyday life tasks affects movement speed. Second, how this effect is compared between PD patients treated with medication or subthalamic deep brain stimulation.

**Methods:**

We used the Card Arranging Reward Responsivity Objective Test that shares component processes with everyday life tasks to compare reward responsivity of movement speed in 10 PD patients with STN-DBS, 10 nonoperated medicated PD patients, both OFF and ON their usual medications/stimulation, and 11 age-matched healthy controls.

**Results:**

Despite longer disease duration and more severe motor symptoms, STN-DBS PD patients with the stimulator turned ON showed greater improvement of movement speed with the prospect of monetary incentive compared to both medicated PD patients and healthy participants.

**Discussion:**

The effect of monetary incentive on movement speed in PD patients is more pronounced with STN-DBS than dopaminergic medications, suggesting that motivational modulation of movement speed may be enhanced as a direct consequence of STN stimulation.

## 1. Introduction

Motivational factors are known to influence motor behaviour in Parkinson's disease (PD), as evident in extreme situations of emotional and physical arousal/stress associated with improved mobility through the phenomenon known as paradoxical kinesis [1–3]. There is also supporting laboratory evidence for the motivational impact of monetary incentive on movement initiation speed, as both PD patients and healthy participants improve reaction times when offered small monetary incentive [4–6]. Nevertheless, little is known about motivational modulation of movement speed beyond life-threatening situations characteristic of paradoxical kinesis or the strict experimental conditions of reaction time studies. Specifically, it is unclear if motivational modulation of movement speed has an impact on bradykinesia in PD in common real-life situations and how this may be affected by various treatments. In the present study, we used a psychomotor task, the Card Arranging Reward Responsivity Objective Test (CARROT) [7] to compare the effect of monetary incentive on movement speed between PD patients with STN-DBS, nonoperated PD patients on dopaminergic medication and age-matched healthy participants.

## 2. Methods

### 2.1. Participants

We studied 10 PD patients with bilateral deep brain stimulation of the subthalamic nucleus (STN-DBS PD: 9 male, mean age 58, range: 39–78), 10 nonoperated PD patients treated with dopaminergic medications (MED PD: 6 male, mean age 60.5, range 50–70), and 11 age-matched healthy participants (5 male, mean age 61, range: 51–70). None of the patients had pathological gambling or other impulse control disorders, as assessed by the question related to dopamine dysregulation syndrome of MDS-UPDRS scale (Question 1.6). The clinical characteristics of the participants are given in Table 1. The study was approved by the local ethics committee, and written informed consent was obtained from all participants.

### 2.2. Experimental Design

PD patients were studied in the OFF and in the ON conditions, on 2 occasions separated by a week. For the OFF condition, MED PD and STN-DBS PD were studied after overnight withdrawal of medications, and in addition, STN-DBS PD patients had the stimulator turned OFF. For the ON condition, medicated PD patients took their usual dopaminergic treatment, while STN-DBS PD patients were studied with the stimulator turned ON, but without medications, in order to capture isolated effects of DBS. To control for potential familiarisation with the task, the healthy participants also completed the experiment twice. In PD patients, the order of ON and OFF sessions was counterbalanced, with half of the patients within each PD group being first tested in OFF and the other half in the ON state.

The severity of motor symptoms in PD patients was assessed with the motor section of the Unified Parkinson's Disease Rating Scale (MDS-UPDRS) [8], in OFF and ON conditions (Table 1). Participants were screened for depression, apathy, and cognitive impairment using the Beck Depression Inventory (BDI) [9], the Marin Apathy Scale (MAS) [10], and the Mini Mental State Examination (MMSE) [11], respectively (Table 1).

### 2.3. Experimental Task

The Card Arranging Reward Responsivity Objective Test (CARROT) is a psychomotor task designed to measure incentive motivation, and it quantifies the extent to which participants increase speed of card sorting when offered a small financial incentive [7, 12–14]. Participants are given a stack of cards, each showing five single digits between 1 and 9 (one number in each corner and one number in the centre), of which one of them is 1, 2, or 3. The aim of the task is to sort cards as quickly as possible into stacks of 1, 2, and 3 piles on whether one of the numbers on the card is 1, 2, or 3. The participants completed three trials. The first was a baseline trial (T1) in which the participant was required to sort 60 cards as quickly as possible, to measure individual baseline speed. For trials T2 and T3, a stack of 100 cards was provided. In T2, the instruction was to sort cards as rapidly as possible within the individualised time limit for each participant measured in T1. T3 was the rewarded trial, and the participant was told that he/she would receive a 10p reward for every five cards sorted, with a 10p coin placed on the table in full view after every fifth card. The participants were not told in advance that they would be offered a reward in the third trial. Time was measured by experimenter with a stopwatch. The number of cards sorted in T2 indicates nonrewarded speed (NRSPEED), while the number of cards sorted in T3 indicates rewarded speed (REWSPEED). The reward responsiveness index (RRI) measures any increment of REWSPEED relative to NRSPEED, that is, RRI = REWSPEED−NRSPEED.

### 2.4. Statistical Analysis

One-way ANOVAs were used to test differences in age distribution and differences on MAS, BDI, and MMSE scales between the 3 groups of participants. To compare UPDRS scores, we used repeated measures ANOVA (rmANOVA), with the between-subject factor PD group (STN-DBS PD vs. MED PD) and the within-subject factor condition (OFF vs. ON). To assess if the repetition of the task in HP affected performance, we performed rmANOVA, with two within-subject factors: session (1st vs. 2nd) and reward (NRSPEED vs. REWSPEED). To assess differences between groups in RRI, we used ANOVAs with the between-subject factor group (3 levels: STN-DBS PD vs. MED PD vs. HP) and the within-subject factor condition, which was for PD patients OFF vs. ON and for HP 1st vs. 2nd session. Post hoc Tukey tests with corrections for multiple comparisons were used to further analyse significant main effects or interactions. The associations between demographic data, clinical motor scores, BDI, MAS, and MMSE on the one hand and RRI on the other hand were examined with Pearson correlations.

## 3. Results

### 3.1. Clinical Scales

There was no difference in age between the two groups of PD patients and healthy participants (*F* (2, 28) = 0.5; *p*=0.61). Disease duration was significantly longer (*p* < 0.001) in STN-DBS PD compared to MED PD. As expected, both groups of PD patients had higher total motor UPDRS in the OFF vs. ON conditions (*F* (1, 19) = 67 *p* < 0.001). Moreover, STN-DBS PD patients had a higher total UPDRS score compared to MED PD patients both in OFF and ON conditions, as revealed by the significant factor group (*F* (1, 19) = 4.7 *p*=0.04), but the nonsignificant group *x* condition interaction (*F* (1, 19) = 0.5; *p*=0.5). For MAS, ANOVA revealed a significant effect of the factor group (*F* (2, 28) = 4.1; *p*=0.027), due to higher apathy scores in STN-DBS PD compared to HC (*p*=0.027). For BDI, the ANOVA revealed significant effect of the factor group (*F* (2, 28) = 3.4; *p*=0.05), due to higher BDI scores in MED PD vs. HCs (*p*=0.04). There was no difference in MAS and BDI between STN-DBS PD and MED PD (*p*=0.13 and *p*=0.5, respectively).

### 3.2. The CARROT

All but one patient (one MED-PD patient in the OFF state) completed both assessment sessions. For each group, the time taken to sort 60 cards in T1, the mean number of cards sorted in the nonrewarded trial T2 and in the rewarded trial T3, and RRI and percentage of improvement in T3 relative to T2 are given in Table 2.

**Figure 1:**
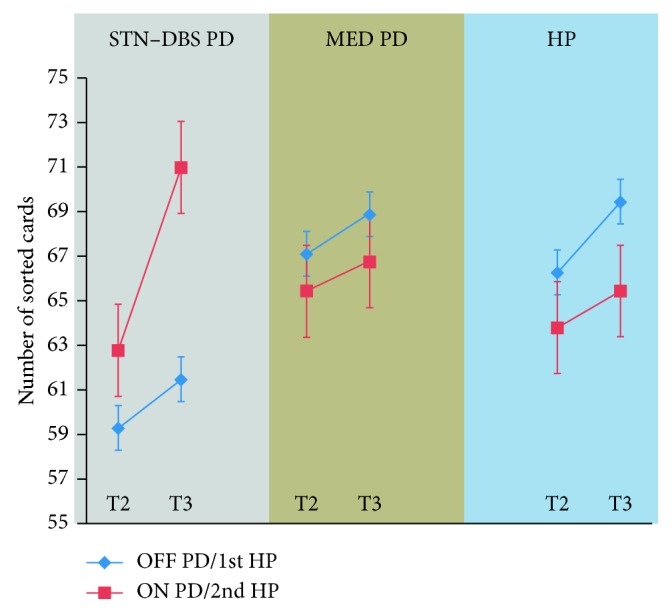
Nonrewarded speed (NREWSPEED) in T2 and rewarded speed (REWSPEED) in T3 are showed for STN-DBS PD patients and MED PD patients in OFF and ON conditions and for first and second experiments for healthy participants. The slope represents RRI, that is, REWSPEED−NRSPEED. PD patients with STN-DBS ON have higher RRI compared to medicated PD patients ON (*p*=0.03) and to healthy participants (*p*=0.03).

For healthy participants, rmANOVA revealed no significant effect of session (*F* (1, 10) = 2.4; *p*=0.16) or reward (*F* (1, 10) = 2.3; *p*=0.15) and no significant 2-way interaction session *x* reward (*F* (1, 10) = 1.3; *p*=0.3), indicating that repeating or familiarisation with the task did not influence the performance. For RRI, ANOVA revealed no significant main effect of the factor group (*F* (2, 27) = 1.8; *p*=0.2) or the factor condition (*F* (1, 27) = 1.2; *p*=0.3), whereas the group *x* condition interaction was significant (*F* (2, 27) =3. 9; *p*=0.03). Post hoc Tukey analysis revealed this was due to higher reward responsiveness in STN-DBS ON vs. MED PD ON (*p*=0.03) and STN-DBS ON vs. HP (*p*=0.03 and *p*=0.03 for STN DBS ON vs. 1st session HP and STN DBS ON vs. 2nd session HP, respectively), while there were no other significant differences (Figure 1).

### 3.3. Correlations

The patients' age, disease duration, UPDRS scores or BDI, MAS, and MMSE scores did not have any noteworthy correlations with RRI.

## 4. Discussion

To study motivational modulation of movement speed in Parkinson's disease, we used the CARROT. This psychomotor task shares strategies with several daily life tasks that require organisation by specific rules, such as sorting clothes by colour for washing, arranging books by topic, or keeping the groceries in the kitchen by compartments. Therefore, the CARROT may be better suited than reaction time experimental paradigms to understand motivational modulation of movement speed that occurs in common life circumstances. Previous studies in healthy participants found that enhancement of speed with monetary incentive on the CARROT correlates with individual differences in appetitive motivation, while in patient populations, the CARROT was shown to be sensitive to change in the motivational state with treatment of apathy [7, 12, 13].

The main result of our study is that STN-DBS PD patients with stimulation turned ON (but no additional dopaminergic medications) improved the movement speed with the prospect of monetary incentive to a greater extent than medicated PD patients and the healthy participants. This effect was present despite longer disease duration and more severe motor impairment for STN-DBS compared to medicated PD patients and despite higher levels of self-reported apathy compared to healthy participants.

The role of the basal ganglia (BG) is to make a selection of movements based on converging information from motor, associative, and limbic circuits [15]. Within BG, STN is a relay nucleus of the indirect pathway and receives direct cortical input via the hyperdirect pathway. Apart from the motor input originating from the motor cortex and the supplementary motor area, the STN receives inputs from associative and limbic cortical and subcortical structures, including the prefrontal cortex, ventral tegmental area, basolateral amygdala, the thalamus, and the ventral *pallidum* [16–20]. The information transmitted through the cortico-STN hyperdirect pathway reaches the basal ganglia output structures before information translated through the direct and indirect corticostriatothalamocortical pathways, suggesting that one of the role of the STN may be in integrating various associative and limbic information related to motor behaviour, before the final output for motor action is sent out from the basal ganglia [21]. In this view, the STN serves as a node to translate motivation into motor action, by processing limbic information that influences motor behaviour [20]. Several studies have reported behavioural changes after STN DBS in patients with PD, and there is evidence to support that these are derived from modulation of limbic-processing neurons within the STN [22–25]. STN DBS in PD has been associated with emergence of explosive-aggressive behaviour [26, 27], mania, and hypomania [28, 29], while accidental lesions of the STN may result in various symptoms of behavioural hyperactivity such as hypersexuality, eurphoria, and impulsivity [20, 30–32]. There is also neurophysiological evidence to support alteration of the limbic and associative circuits following STN DBS. In PD patients, 18F-FDG PET (performed before and 3 months after surgery) showed metabolic changes in several cortical regions that are part of limbic and associative circuits [33, 34]. Using intraoperative fMRI during high frequency STN stimulation in PD patients, blood oxygen level-dependent signal changes were observed not only in the motor circuitry but also in the limbic circuitry, including cingulate and insular cortices [35].

The results of the present study add to the line of evidence linking STN-DBS or STN lesions (which are assumed to have roughly similar inactivation effects as stimulation of the hyperactive STN in human PD) to heighten incentive motivation [36–38]. For example, increased sensitivity to food reward cues associated with postoperative weight gain has been documented after STN-DBS in PD patients [39, 40]. We have previously shown in the same group of PD patients that monetary incentive improves reaction times irrespective of patients being off or on medication or STN-DBS [5, 6]; however, only patients treated with STN-DBS (with stimulation turned on) were capable to further improve initiation time with higher reward magnitude, suggesting enhanced incentive motivation as a result of STN stimulation [6]. Interestingly, our STN-DBS PD patients with stimulation ON showed relatively larger improvement of movement speed with reward than healthy participants. One explanation is the “ceiling effect,” as healthy participants could have already reached their near to maximal speed in the nonrewarded trial (note that instructions for the nonrewarded trial were to sort out cards as quickly as possible). Percentage of improvement in the rewarded trial in our group of healthy participants was around 4% which is in line with previous studies on healthy subjects [13].

Our results show dissociation between the deficient motivation represented by self-reported apathy (as measured by MAS) and experimental modulation of movement speed in response to small monetary incentive. This contra-intuitive effect may be possibly related to impulsivity. Some animal experimental studies suggest that higher reward sensitivity in STN-lesioned animals is associated with increased impulsivity [36, 37, 41] and studies in PD patients using the STN DBS ON vs. OFF methodology found that STN DBS in PD patients is associated with inhibitory deficit over anticipatory responses [6, 42]. Nevertheless, as our study was not designed to monitor anticipation errors, we cannot provide evidence to support the latter hypothesis.

## 5. Study Limitation

The main limitation of the study is the relatively small number of participants in each group. However, use of a repeated measures design allowed us to detect within-subject changes of movement speed between nonrewarded and rewarded trials in different motor conditions (ON vs. OFF medication or stimulation), increasing the statistical power. A repeated measures design may, however, be a source of a potential bias, since the participants repeated the CARROT twice and thus became familiarised with the task. Since in the second session participants knew they would be performing a rewarded trial, hypothetically they could strategically slow their performance on the nonrewarded trial in order to improve more in the rewarded trial. However, we believe that repetition of the task did not affect the results. First, we did not detect any differences between nonrewarded and rewarded trials in first and second sessions in healthy participants. Second, we counterbalanced the ON and OFF conditions in PD patients. Finally, there are previous studies that have successfully used the CARROT repeatedly in the same participants, in order to detect the effect of various measures on reward responsiveness [13, 14].

## 6. Conclusions

We have demonstrated, using a psychomotor CARROT task, that PD patients with STN-DBS ON (and no dopaminergic medications) showed greater improvement of movement speed with the prospect of monetary incentive compared to medicated PD patients and age-matched healthy participants. This suggests that motivational modulation of movement may be enhanced and be directly related to STN stimulation. This finding may be relevant for incorporating reward cues into rehabilitation programmes for patients after STN-DBS treatment.

## Figures and Tables

**Table 1:** Clinical characteristics of participants.

	STN-DBS PD	MED PD	HP
Disease duration (years)	14 (1.5)	4.8 (1.6)	—
Total motor UPDRS	OFF 41.6 (4.9)	OFF 30.4 (3)	—
	ON 21.9 (2.3)	ON 15.3 (2.1)	
LED	320 (40)	393 (44)	—
MAS	38.5 (2.2)	32.2 (2.8)	30.1 (1.5)
BDI	8.8 (1.2)	10.7 (1.4)	5.8 (1.6)
MMSE	29.1 (0.5)	29.5 (0.2)	29.8 (0.1)

Data are given as a mean and standard error within the brackets. Abbreviations: STN-DBS PD, PD patients on STN DBS; MED PD, medicated PD patients; UPDRS, Unified Parkinson Disease Scale; LED, L-Dopa Equivalent Dose in milligrams; MAS, Marin Apathy Scale; BDI, Beck Depression Inventory; MMSE, Mini Mental Status Examination.

**Table 2:** Number of sorted cards in Trials 1, 2, and 3, reward responsivity index, and percentage of improvement with rewarded trial.

		T1	T2	T3	RRI	% of improvement^*∗*^
STN-DBS PD	OFF	128.2 (20.3)	59.3 (3.8)	61.5 (3.4)	2.2 (2.3)	4.8 (4.3)
	ON	87.2 (8.5)	62.8 (2.1)	71 (2.6)	8.2 (1.6)	13.3 (2.8)

MED-PD	OFF	72.8 (5.8)	67.1 (1.9)	68.8 (2)	1.8 (1.2)	2.7 (1.9)
	ON	68.5 (3.6)	65.4 (1.9)	66.7 (1.6)	1.3 (1.4)	2.3 (2.2)

HP	First	55.2 (3.7)	66.2 (1.4)	69.4 (2.1)	3.1 (1.3)	4.6 (1.9)
	Second	53.5 (4.2)	63.8 (1.3)	68.5 (1.5)	2 (1.8)	3.2 (2.9)

Data are given as a mean and standard error within the brackets. Abbreviations: STN-DBS PD, PD patients on STN DBS; MED PD, medicated PD patients; T, trial; RRI, reward responsivity index; ^*∗*^% of improvement in the rewarded trial relative to the nonrewarded trial.

## Data Availability

The data used to support the findings of this study are available from the corresponding author upon request.
